# CHRM3-Associated miRNAs May Play a Role in Bile Acid-Induced Proliferation of H508 Colon Cancer Cells

**DOI:** 10.5152/tjg.2022.22605

**Published:** 2023-03-01

**Authors:** Çağdaş Aktan, Fatih Tekin, Nevin Oruç, Ömer Özütemiz

**Affiliations:** 1 Department of Medical Biology, Beykent University Faculty of Medicine, İstanbul, Turkey; 2 Department of Gastroenterology, Ege University Faculty of Medicine, İzmir, Turkey

**Keywords:** hsa-miR-1246, hsa-miR-522-3p, colon cancer, bile acid, muscarinic receptor 3, microRNA

## Abstract

**Background:**

It was well defined that proliferative effects of bile acids on colon epithelium are through interaction with muscarinic-3 receptors. Recently, microRNA emerged as an important regulator of gene expression and has been implicated in pathogenesis of many malignancies. However, the interaction of *CHRM3* and microRNAs and their potential effects on colon carcinogenesis remains to be elucidated.

**Methods:**

In the current study, analysis of cell proliferation for 6 days after treatment with sodium taurolithocholate was analyzed by using WST-1 method. microRNAs which possibly target *CHRM3* were identified by in silico analyses. Expression profiling of these microRNAs, expression changes of *CHRM3* gene at mRNA level for H508 and SNU-C4 colon cancer cells were analyzed by quantitative polymerase chain reaction; the protein level of CHRM3 was analyzed using Western blot; apoptotic experiments were analyzed using the Annexin V assay. The Gene Ontology and the Kyoto Encyclopedia of Genes and Genomes pathway enrichment analyses were performed using the miRPath v3.0.

**Results:**

It was found that the expression level of *CHRM3* gene was 6.133 ± 0.698-fold in H508 cells compared with SNU-C4 cells (*P* =.004). Treatment of H508 cells with sodium taurolithocholate caused 1.34 ± 0.4156-fold change in the expression level of *CHRM3* gene (*P* =.0448). No apoptotic changes were observed in both colon cancer cells after treatment with sodium taurolithocholate. Different expression changes were detected of hsa-miR-129-5p, hsa-miR-30c-5p, hsa-miR-224-5p, hsa-miR-30b-5p, hsa-miR-522-3p, and hsa-miR-1246. Finally, hsa-miR-1246 and hsa-miR-522-3p could play a critical role in tumor development via bile acid-related genes in colon cancer.

**Conclusion:**

These findings reflected that *CHRM3*-dependent oncogenetic pathways might be in charge of colon cancer. We suggest that the microRNA expression profile of each individual colon cancer tissue is a unique digital signature.

**Main Points**

- *CHRM3*-dependent oncogenetic pathways might be in charge of colon cancer.
- Expression of *CHRM3*-dependent hsa-miR-1246 and hsa-miR-522-3p could play a critical role in tumor development via bile acid-related genes in colon cancer.
- microRNA expression profile of each individual colon cancer tissue is a unique digital signature.

## Introduction

Colorectal cancer accounts for 9.4% of cancer deaths and 10% of global cancers. It is estimated that the incidence of colorectal cancer will increase further in the coming years. This increase in the incidence of the disease is mainly due to environmental and genetic factors such as diet, lifestyle, and mutations in various genes.^1^

Bile acids (BAs) are a class of steroid acids that are abundant in humans and are produced from cholesterol metabolism in the mammalian liver. After BAs are synthesized in the liver, they are stored in the gallbladder and released into the small intestine. Following intestinal transit, most BAs are reabsorbed into the hepatic portal vein in the terminal ileum and transported back to the liver.^2^

There are various studies investigating the role of BAs in the progression of colon cancer. BAs have been shown to promote the proliferation of colon epithelium in animal models and human colonic cell lines.^3^ The fecal excretion of total BAs, deoxycholic acid, and lithocholic acid has been found to be higher in patients with colon cancer and adenomas compared to normal controls.^4,5^ Furthermore, increased risk of right-sided colon cancer in patients who had undergone cholecystectomy has been well described in epidemiological studies.^6^ Bile flow into the colon increases in patients with cholecystectomy since there is no pooling of bile in the gallbladder. Previous in vitro studies using colon cancer cell lines have shown that the proliferative effects of BAs on the colon epithelium are through interaction with muscarinic-3 receptors (CHRM3).^7,8^

microRNAs (miRNAs) belong to the class of non-coding RNAs with a length of 22-24 nt (nucleotide) and can change the expression levels of the mRNAs. These non-coding miRNAs involve in multiple events including the development of colon cancer and tumor progression. It has been shown that alterations in miRNAs expression are involved in the invasion and metastasis of colon cancer.^9^ However, the mechanisms of miRNAs in colon cancer development have not been understood clearly. In addition, the interaction of BAs with both CHRM3 and *CHRM3*-targeted miRNAs and their potential effects in colon carcinogenesis remains to be elucidated. This study aims to investigate the possible role of *CHRM3*-targeted miRNAs using 2 human colon cancer cell lines: H508 strong-expressing *CHMR3* and SNU-C4 weak-expressing *CHRM3*.

## Materials and Methods

### Cell Culture and Reagents

H508 (CCL-253) and SNU-C4 (Cat# 0000C4) human colon cancer cells were purchased from ATCC and Korean Cell Line Bank (http://cellbank.snu.ac.kr-0000C4), respectively. These cells were maintained in Roswell Park Memorial Institute (RPMI)-1640 medium containing 10% heat-inactivated fetal bovine serum (FBS), 100 units of penicillin-streptomycin/mL, and 1% l-glutamine, using 75 cm^2^ flasks at 37°C with 5% CO_2_ in a humidified air. When the cells reached enough confluency, they were harvested from a cell culture flask using trypsin-Ethylene diamine tetraacetic acid (EDTA) solution and used for further experiments. Sodium taurolithocholate (ST) (T7515) was purchased from Sigma Aldrich. Stock solutions of ST (300 µM) were prepared using methanol.^8,10^

### Cell Proliferation Assay

The cellular proliferation of H508 and SNU-C4 cells was analyzed using a colorimetric WST-1 Cell Proliferation Reagent (Roche, Germany) according to the manufacturer’s protocol. The cells were seeded in 96-well plates (Corning, New York, USA) at approximately 10% confluency with growth medium and allowed to attach to plate bottom for 24 hours. After 24 hours incubation at 37°C in the humidified incubator with 5% CO_2_, growth medium was removed from 96-well plates and fresh FBS-free medium containing different concentrations of ST was added to each well and then incubated at 37°C in a humidified incubator with 5% CO_2_. The cells were treated with 1:10 WST-1 Cell Proliferation Reagent every day for 5 days to find an appropriate cell proliferation concentration. Absorbance values of samples at 450 nm and 620 nm (reference) wavelengths were measured using the SpectraMax 384 Plus plate reader (Molecular Devices, USA).

### Quantitative Real-Time Polymerase Chain Reaction

Total RNA was extracted from the cells, which have been treated with ST and untreated, using miRNeasy Mini Kit (Qiagen, Germany), according to the manufacturer’s instructions. cDNA was synthesized from total RNA using All-in-One First Strand cDNA Synthesis Kit for gene quantitative polymerase chain reaction (qPCR) array (Genecopoeia, USA) and All-in-One miRNA First Strand cDNA Synthesis Kit for miRNA qPCR array (Genecopoeia, USA). Quantitative real-time PCR (RT-qPCR) was performed using All-in-One™ qPCR Mix (Genecopoeia, USA) and appropriate primers using Applied Biosystems ABI 7500 Fast RT-PCR system. Glyceraldehyde-3-phosphate dehydrogenase (*GAPDH)* and *β-actin* were used as endogenous controls for normalization of *CHRM3* gene expression.

The “miRWalk” (http://zmf.umm.uniheidelberg.de/apps/zmf/mirwalk2/) database was used to determine appropriate miRNAs. Twenty-five miRNAs were chosen for the qPCR array based on their potential roles in colorectal cancer progression and targeted to *CHRM3* gene. The specifically designed and synthesized primers for the miRNAs were adapted to Applied Fast Real-Time PCR using All-in-One qPCR Mix (Genecopoeia, USA). The miRNA qPCR array also included *SNORD61, SNORD68, SNORD72, SNORD95,* and *RNU6-2* endogenous controls for normalization of the miRNAs expression. All raw threshold cycle (Ct) values were calculated using SDS software v.2.1 of qPCR machine setting baseline and threshold.

### Western Blot Analysis

The proteins were extracted in 100 µL of Complete Lysis-M Buffer (Roche, Germany) kit following the instructions. Total protein concentration was calculated using the Bradford method. About 40 µg/well of each protein extract was transferred onto a polyvinylidene difluoride (PVDF) membrane following electrophoresis at 8% Sodium Dodecyl Sulphate-Polyacrylamide Gel Electrophoresis (SDS-PAGE) gel using iBlot Blotting System (Invitrogen, USA). Concentration of the primer antibody was 1:1000 diluted CHRM3 (Abcam-ab126168, Germany) and β-actin (Cell Signaling-#4970, Germany) antibodies. iBlot Western Detection Kit (Invitrogen, USA) was used in incubation, blotting, and washing steps. Signals were detected using chromogenic substrate and imaged using a gel imaging system.

### Apoptosis Analysis

The apoptotic effect was quantified on H508 and SNU-C4 cells after 5 days treatment with a 300 µM concentration of ST. For this purpose, 1 × 10^5^ cells per well were seeded in 6-well plates. The groups that did not include ST were evaluated as the control group. The cells were washed with PBS and harvested after 5 days. Apoptosis analysis was performed using the Annexin V FITC/propidium iodide Apoptosis Detection kit (BioVision, Germany). The number of apoptotic, necrotic, and living cells was calculated using a flow cytometer (BD Biosciences, Germany).

### Pathway Enrichment Analysis

miRPath v3.0 (https://dianalab.e-ce.uth.gr/html/mirpathv3/) was used for pathway enrichment analysis of the identified miRNA set. To better understand the mechanisms underlying colon carcinogenesis related to *CHRM3* gene, the Gene Ontology (GO) and the Kyoto Encyclopedia of Genes and Genomes (KEGG) pathway enrichment analyses were conducted using differentially expressed miRNAs (DEMs) via miRPath v3.0. This tool assigns the GO and KEGG pathways with significance level determined by the number of target genes affected by the identified miRNAs.

### Statistical Analysis

The log-fold change of *CHRM3* gene and each miRNA was calculated from the delta Ct (ΔCT) levels between cells treated with ST and untreated cells using the delta-delta Ct (ΔΔCT) method. The expressions of the *CHRM3* gene and miRNAs in the groups were compared with unpaired *t*-test with Welch’s correction and Student’ s *t*-test, respectively. Images of western blot were analyzed using ImageJ software (ImageJ 1.53o (https://imagej.nih.gov/ij/). The statistical analyses were performed using GraphPad Prism software version 7.0 for Windows (GraphPad Software, San Diego, Calif, USA) and Statistical Package for Social Sciences (SPSS) version 25 for Windows software package (IBM Corp.; Armonk, NY, USA). All data unless otherwise indicated are shown as mean values ± standard error of the mean (SEM). The GO and KEGG pathway analyses were identified by a posteriori statistical analysis, selecting Union of Pathways, an embedded option in the DIANA miRPath v.3 web-based computational tool. A *P* value of <.05 was considered statistically significant.

## Results

### *CHRM3* Gene Expression

**Figure 1.**
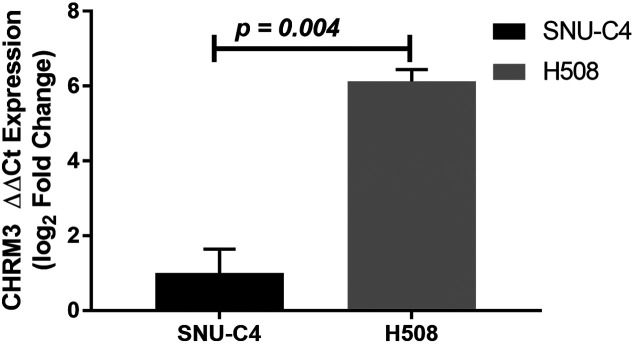
Relative expression level of *CHRM3* for H508 cells compared with SNU-C4 cell. The relative expression level of the *CHRM3* in each cell was measured by RT-qPCR, and was normalized by the expression level of *GAPDH *and *β-actin*. Statistical analysis was performed with Student’s *t*-test. Values represent mean ± SEM (n = 3). RT-qPCR, real-time quantitative polymerase chain reaction; SEM, standard error of mean.

The expression level of *CHRM3* gene was upregulated in H508 cells compared with SNU-C4 cells (Figure 1). It was observed that *CHRM3* gene expression level was 6.133 ± 0.698-fold in H508 cell compared with SNU-C4 cell (*P* =.004).

### Cell Proliferation

**Figure 2.**
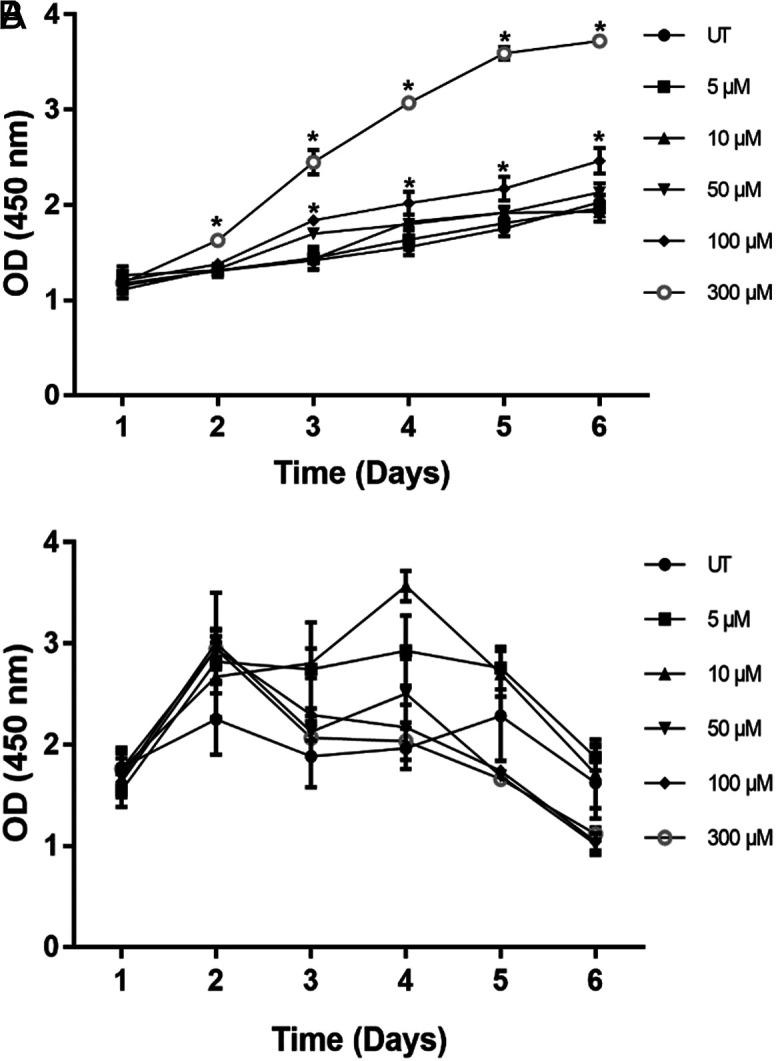
(A) Effects of ST on the proliferation of H508 and (B) SNU-C4 human colon cancer cells. Time course for the effects of increasing concentrations of ST on the proliferation of the cells. Statistical analysis was performed with 2-way ANOVA with Tukey’s post hoc test for multiple comparisons. Values represent mean ± SEM (n = 3). *P <.0001. ANOVA, analysis of variance; SEM, standard error of mean; ST, sodium taurolithocholate.

In the proliferation assay, changes in proliferation rate of H508 and SNU-C4 cells were observed for 5 days after treating cells with ST. Concentrations of 5, 10, 50, 100, and 300 μM ST were used in each well to estimate the most effective ST concentration on cell proliferation. The experiments for each cell lines were performed in triplicates and compared with untreated cells (UT). A significant increase in cell proliferation with 100 and 300 μM concentrations of ST was detected at third and second days, respectively (*P* < .0001). Furthermore, a significant increase was detected with 100 and 300 μM concentrations of ST in cell proliferation at the fourth, fifth, and sixth days (*P* < .0001). It was determined that the proliferative effects of 300 μM concentration of ST reached a plateau phase after the fifth day, and there was an approximately 3-fold increase in proliferation. Therefore, it was determined that 300 μM concentration of ST was appropriate in the H508 cells, and this concentration was also used in the SNU-C4 cells. Since gene expression of *CHRM3* was not observed abundantly in the SNU-C4 cells because of knocking-down of this gene, minimal proliferation was observed in this cell line (*P* <.05) (Figure 2).

**Figure 3.**
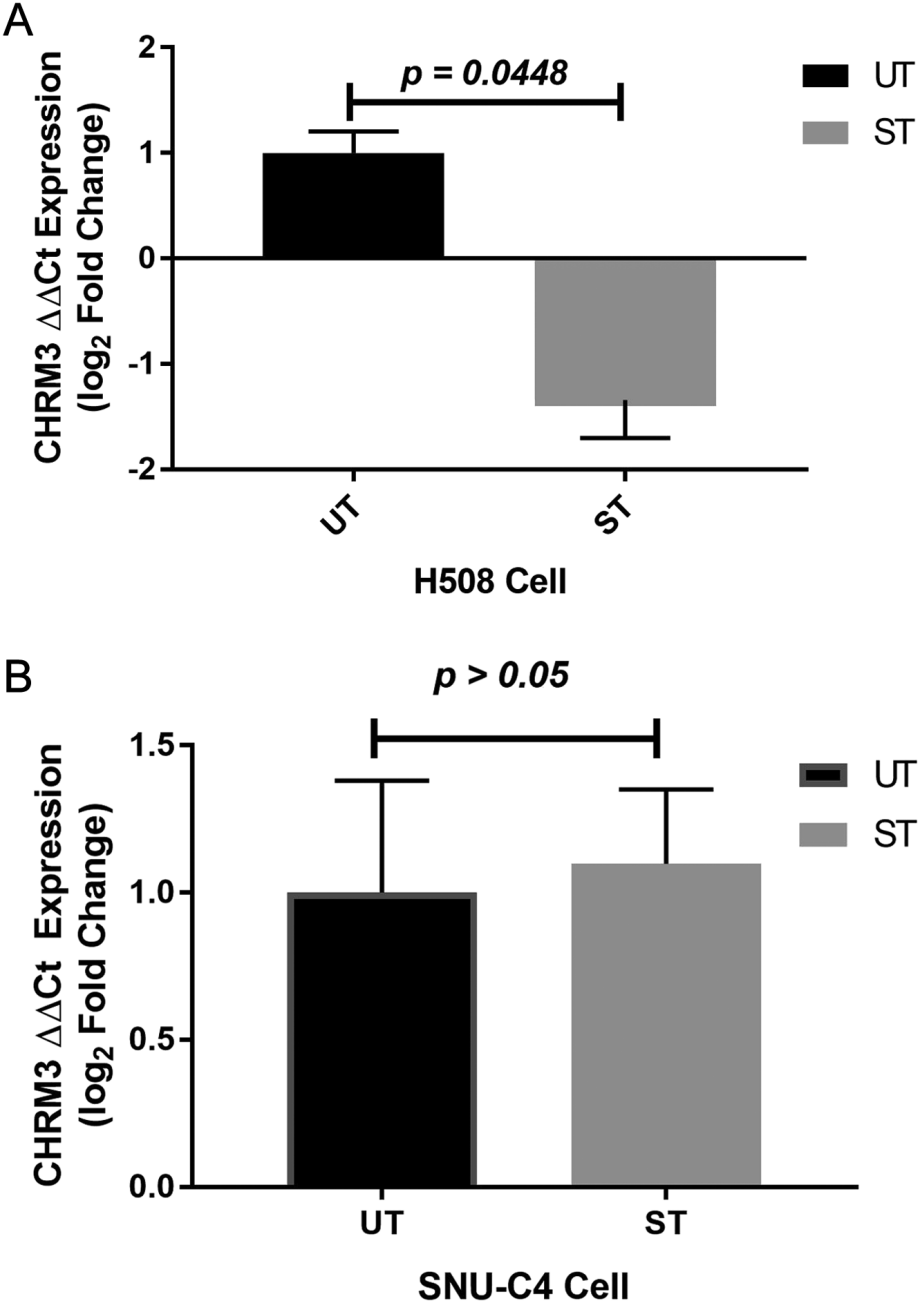
(A) Effects of ST on *CHRM3* gene expression of H508 and (B) SNU-C4 human colon cancer cells. The expression levels of *CHRM3* mRNA expression in response to the ST treatment compared with untreated (UT). The expression of the *CHRM3* level was measured by RT-qPCR. It was normalized by the expression level of *SNORD61, SNORD68, SNORD72, SNORD95,* and *RNU6-2*. Statistical analysis was performed with Student’s *t*-test. Values represent mean ± SEM (n = 3). RT-qPCR, real-time quantitative polymerase chain reaction SEM, standard error of mean; ST, ST, sodium taurolithocholate.

To determine the effect of ST on *CHRM3* gene expression in H508 and SNU-C4 cells, the experimental set was established by treating concentration of 300 μM ST, which was determined in cell proliferation analysis. Treatment of H508 cells with ST caused 1.34 ± 0.4156-fold change of *CHRM3* gene expression (*P* = .0448), whereas treatment of SNU-C4 cells with ST caused 1.098 ± 0.4569-fold of *CHRM3* gene expression but the difference was not significant (*P* > .05) (Figure 3).

### Alteration in micro RNAs Expression That Potentially Regulates *CHRM3* Gene

To investigate the role of miRNAs in proliferation mechanism of colon cancer cells, the expression changes of 25 candidate miRNA molecules targeting *CHRM3* gene were analyzed in H508 and SNU-C4 cells. For this purpose, the cells treated with ST were compared to the UT cells in both cell lines. The experiments were performed in 3 replicates for each cell lines.

**Figure 4.**
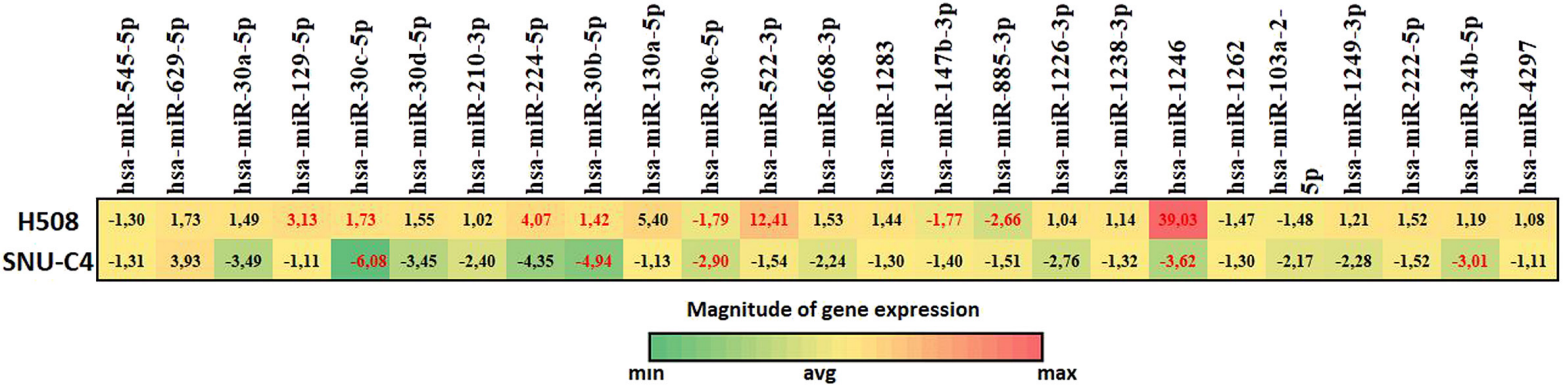
Effects of ST on miRNA expression compared with untreated (UT) H508 and SNU-C4 colon cancer cells. The magnitude of gene expression bar and the color range of minimum, maximum, and average fold change are shown in the bottom (red = upregulation; green = downregulation). The relative expression level of the miRs in each sample was normalized by the expression level of *SNORD61, SNORD68, SNORD72, SNORD95,* and *RNU6-2*. Statistical analysis was performed with Student’s *t*-test. Values represent fold change. miRNA, micro RNA; ST, sodium taurolithocholate.

There were 10 miRNAs that exhibited significant differences in expression between H508 and SNU-C4 cells (*P* <.05); among them, 6 upregulated and 3 downregulated miRNAs were determined in H508 cells and 5 downregulated miRNAs in SNU-C4 cells. Upregulated miRNAs in H508 cells included hsa-miR-129-5p, hsa-miR-30c-5p, hsa-miR-224-5p, hsa-miR-30b-5p, hsa-miR-522-3p, and hsa-miR-1246, whereas downregulated miRNAs included hsa-miR-30e-5p, hsa-miR-147b, and hsa-miR-885-3p. On the other hand, downregulated miRNAs in SNU-C4 cells included hsa-miR-30c-5p, hsa-miR-30b-5p, hsa-miR-30e-5p, hsa-miR-1246, and hsa-miR-34b-5p (Table 1 and Figure 4).

### Western Blot and Apoptosis Results

**Figure 5.**
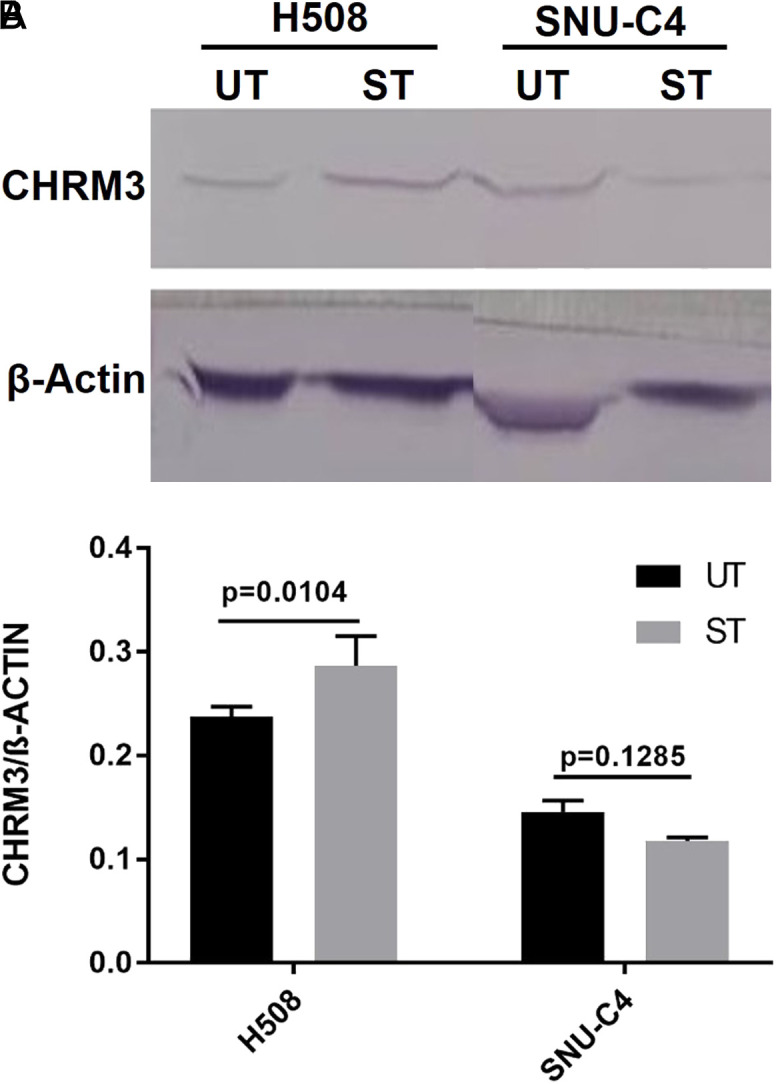
(A) Effects of ST on expression of CHRM3 of H508 and SNU-C4 human colon cancer cells. UT, Untreated. Specific protein bands detected by western blots were quantified using ImageJ software and normalized against β-actin. (B) Relative quantification of Western blot analysis is depicted in the bar graphs. Statistical analysis was performed with 2-way ANOVA with Sidak’s post hoc test for multiple comparisons. Values represent mean ± S.E.M (n = 3). ANOVA, analysis of variance; SEM, standard error of mean; ST, sodium taurolithocholate

**Figure 6.**
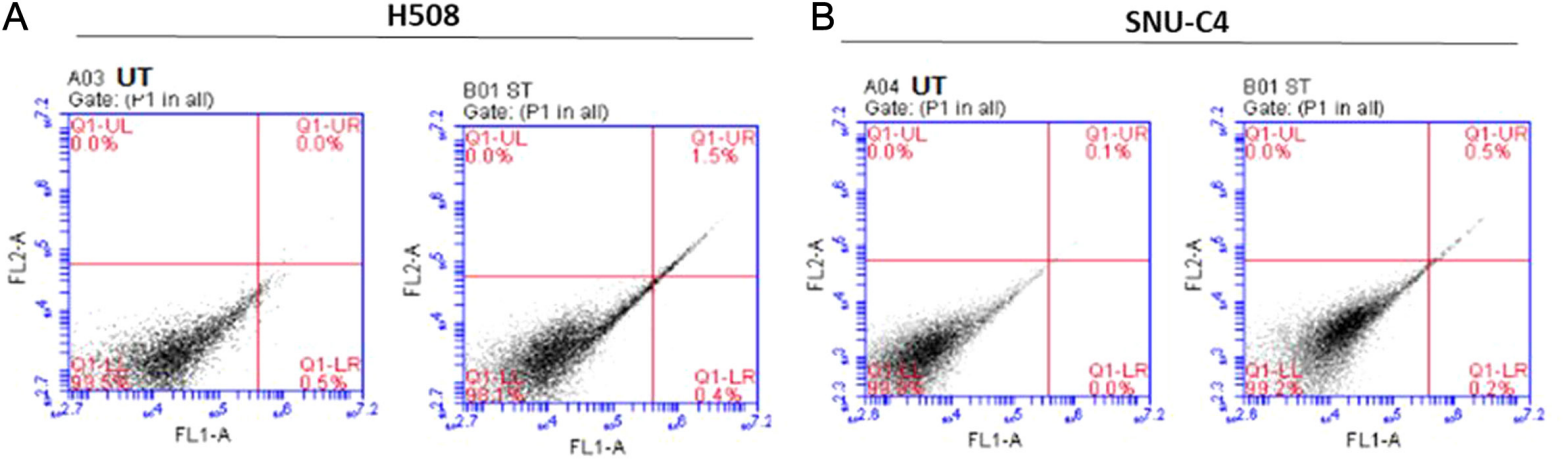
(A) Effects of sodium taurolithocholate (ST) on apoptosis of CHRM3 of H508 and (B) SNU-C4 human colon cancer cells. UT, Untreated. In all experiments, treatment with ST did not trigger apoptosis. The rate of apoptotic cells was calculated by qualitative flow cytometry using Annexin V and propidium iodide (PI) staining. Q1-LL quadrant (Annexin V FITC/PI) is viable cells; Q1-LR quadrant (Annexin V FITC+/PI) is early apoptotic cells; and Q1-UR and Q1-UL quadrants (Annexin V FITC+/PI+) are late apoptotic and necrotic, respectively. LL, lower left; LR, lower right; UL, upper left; UR, upper right. (A) H508 human colon cancer cell. (B) SNU-C4 human colon cancer cell.

At the end of the 5-day waiting period, as a result of Western blot using protein extracted from cells treated with ST, it was determined that CHRM3 protein expression showed a significant change in H508 cells (0.2372 ± 0.01) compared with control (UT) (0.2868 ± 0.02) (*P* =.0104), whereas no statistical change was observed in SNU-C4 cells (0.1456 ± 0.01 and 0.1179 ± 0.002, respectively) (*P* >.05) (Figure 5). In all experiments, treatment with ST did not trigger apoptosis (Figure 6).

### Gene Ontology and Kyoto Encyclopedia of Genes and Genomes Pathway Enrichment Analysis

According to KEGG enrichment analysis, 17 significant pathways of common DEMs were identified, including: “apoptosis,” “pathways in cancer,” “Wnt signaling pathway,” “p53 signaling pathway,” and “colorectal cancer”. The significant genes in these pathways are presented in Table 2. In the present study, 230 up- and downregulated DEMs (of which 128 were significant *P* < .05) were obtained and mainly enriched in “gene expression,” “RNA binding,” “response to stress,” “biological process,” “membrane organization,” “EGFR signaling pathway,” and “cell death.” The GO distribution of the DEMs is presented in Table 2.

## Discussion

*CHRM3* gene can play a conditional oncogenic role, because its overexpression stimulates the cell functions such as proliferation, invasion, and apoptosis in colon cancer.^10^ The relationship between the expression level of *CHRM3* and the anatomic location, stage, and differentiation of colon cancer was investigated in previous studies and no statistically significant difference was observed between the groups.^11^ However, the right-sided colon tumors are more prone to bile acid. It has been reported that BAs can induce oncogenesis and tumor progression via *CHRM3*-mediated pathways. However, studies investigating the roles of miRNAs in this pathogenic pathway are limited.^11^ To test this hypothesis, we selected 2 different colon cancer cell lines SNU-C4 and H508 with negative and high *CHRM3* gene expression, respectively.^7^ We investigated the expression of miRNAs detected using in silico methods in these cell lines.

BAs stimulate the proliferation of colon cancer cells. Cheng et al^12^ has revealed that BAs stimulate proliferation of H508 colon cancer cells which co-express both *CHRM3* and *EGFR*. On the other hand, cell proliferation was not stimulated by BAs in CHO colon cancer cells that express only *CHRM3* and SNU-C4 cells that express only *EGFR*. These results show that bile acid-induced colon cancer cell proliferation is dependent on *CHRM3* and is mediated by signal transduction of several molecules such as *EGFR* transactivation and Erk1/2.^12^ In our experimental settings, the results of cell proliferation induced by bile acid were similar to the results of previous studies. In our settings, BAs have induced proliferation patterns only in H508 cells, which express *CHRM3*. Although we could not measure the expression of *EGFR, CHRM3* expression level remains stable even after treating the H508 cells with bile acid.

BAs are known as molecules that have toxic effects on colonic cells. They induce apoptosis as a result of several mechanisms such as release of cytochrome *C*, Reactive oxygen species (ROS) formation, and cytosolic caspase activation.^13,14^ After exposure of BAs to colon epithelial cells at high concentrations, it strongly induces cell death, while at normal physiological concentrations inhibits the degradation of the *p53* gene and as a result becomes resistant to apoptosis.^15,16^ Thus, these findings indicate how BAs promote colon cells to cancer cells or resistance of colon cancer to chemotherapy and radiation. In our study, BA was used at physiological concentration, and obtained an increased rate of proliferation, but resistance to apoptosis in H508 cells.

Previous studies have reported that different miRNA expression profiles in colon adenocarcinoma compared to its normal adjacent tissues.^17^ However, there is no published study investigating the interaction of BAs with both CHRM3 and *CHRM3*-targeted miRNAs and their potential effects in colon carcinogenesis. We demonstrated different miRNA expression levels in H508 and SNU-C4 cells after treating them with ST bile acid. One of them, hsa-miR-1246 is known to be important in progression of several cancer types, including colon cancer. It was found to be highly expressed in progressive colon tumors.^18^ It is also suggested as a biomarker for early detection of certain malignancies. It functions as a part of the *p53*-associated cell-cell network, which plays an important role in many cancers such as colon cancer.^18^ It has also been reported that this miRNA increases metastasis and plays a role in the mechanisms of chemical resistance and self-renewal ability in colorectal cancer.^19,20^ It was upregulated in colon cancer tissue and serum compared with their controls.^21^ All these findings have suggested that hsa-miR-1246 might be a potential biomarker of colon cancer. In our in vitro study, we found that hsa-miR-1246 was the most upregulated miRNA after treatment with ST. After ST treatment, a significant upregulation of hsa-miR-1246 expression by 39.03-fold in H508 cells, which were known to express *CHRM3*, was observed, whereas it resulted in significant downregulation by 3.62-fold in SNU-C4 cells. This result highly suggests that ST induces hsa-miR-1246 expression through binding or activating to *CHRM3*. Further, we might speculate that only cells that express *CHRM3* might go through oncogenesis cycles by induction of ST. This hypothesis might be one of the oncogenetic steps especially in right-sided colon tumors. In our in vitro study, treatment with ST resulted in significant upregulation of hsa-miR-224-5p expression by 4.07-fold in H508 cells, whereas it did not significantly change in SNU-C4 cells.

hsa-miR-522-3p has been shown to be upregulated in some cancers such as glioblastoma and non-small cell lung cancer.^22,23^ It has been reported that this miRNA reverses drug resistance of doxorubicin-induced HT29 colon cancer cell by targeting *ABCB5*. The results suggested that this miRNA might be important in the development of colon cancer chemotherapy resistance.^24^ It has also been reported that *BVES-AS1* lncRNA inhibited colorectal adenocarcinoma progression via interacting with hsa-miR-522-3p.^25^ In our study, hsa-miR-522-3p was induced 12.41-fold after treating with ST in H508 cells. On the contrary, there was no significant change in hsa-miR-522-3p expression after treating with ST in SNU-C4 cells. This result also suggests that hsa-miR-522-3p is another important messenger for oncogenesis though ST induced *CHRM3*-related pathways in colon cancer. Since our study demonstrated that exposure of ST decreases hsa-miR-522-3p absence of *CHRM3* in SNU-C4 cells, we might speculate that its expression is highly related to *CHRM3*.

In our in vitro study, treatment with ST resulted in significant upregulation of hsa-miR-224-5p expression by 4.07-fold in H508 cells, whereas it did not significantly change in SNU-C4 cells. Recent study by Yang et al^26^ reported that miRNA profile differs between right- and left-sided tumors. They showed that hsa-miR-224-5p was 4 times less expressed in right-sided tumors. We might hypothesize that more bile salt exposure and contact to the right side colon might induce oncogenesis only if CHRM3 receptors are present. Yang et al^26^ also obtained 22 optimal diagnostic miRNA biomarkers in right-sided colon tumors compared to left-sided tumors, among which 3 were significantly DEMs (hsa-miR-155-5p, hsa-miR-224-5p, and hsa-miR-31-5p). Those miRNAs might be investigated further in *CHRM3* expressing right- and left-sided colon tumors.

We found that treatment with ST resulted in significant upregulation of hsa-miR-129-5p expression by 3.13-fold in H508 cells, whereas it did not significantly change in SNU-C4 cells. The differential expression among mRNAs, lncRNAs, and miRNAs in LoVo colon cancer cells was compared in a recent study. lncRNA *MALAT1* and mRNA *NFAT5* in LoVo colon cancer cells were significantly upregulated, while miR-129-5p was significantly downregulated.^27^ This finding confirms that miR-129-5p expression is mostly *CHRM3* dependent while it did not significantly change in SNU-C4 cell line in our settings. This finding also suggests that each individual might have a different tumorigenesis fingerprint in the aspect of miRNA expression.

We found that treatment with ST resulted in significant decrease of hsa-miR-885-3p expression by 2.66-fold in H508 cells, whereas it did not significantly change in SNU-C4 cells. Furthermore, hsa-miR-885-3p has been shown to suppress angiogenesis and reduce colon cancer cell growth through disruption of BMPR1A and Smad/Id1 signaling by Xiao et al.^28^ Treatment with ST suppresses this inhibitory miRNA in the presence of *CHRM3* and promotes the cell proliferation.

The expression of hsa-miR-147b slightly decreased by 1.77-fold in H508 cells while it did not change in SNU-C4 cells treatment with ST. Gaechke et al indicated that 49 miRNAs significantly expressed in rectum cancer. However, 21 miRNAs potentially define rectal cancer as a disease molecularly distinct from other colon tumors. The expression of hsa-miR-147b was one of that defining rectum cancer.^29^ Bile acid most likely induces right-sided colon tumors and they decrease the expression of hsa-miR-147b in the presence of *CHRM3* gene in our settings. Since the exposure rate of BAs to the rectum mucosa is possibly low, the expression level of hsa-miR-147b might be higher in right-sided tumors independent from the expression of *CHRM3* gene.

In our study, it was found that treatment with ST resulted in significant downregulation of hsa-miR-30c-5p expression by 6.08-fold in SNU-C4 cells, whereas it resulted in significant upregulation by 1.73-fold in H508 cells. Previously it was reported that low expression of hsa-miR-30c-5p and hsa-miR-223-3p was associated with better survival in the colon cancer. We suggest that in the absence of *CHRM3* expression, BAs have inhibitory effects on hsa-miR-30c-5p expression. Similarly, hsa-miR-30e-5p is a novel effector of p53-induced suppression of migration, invasion, and metastasis in colon cancer.^30^ The expression of hsa-miR-30e-5p decreases by 1.79-fold after ST treatment in H508 cells while decreases 2.9-fold in SNU-C4 cells. The observed differential expression levels of this miRNA in both cell lines confirm that it acts as a tumor suppressor miRNA in all types of colon cancer cells and acts independent from *CHRM3* gene. Furthermore, in KEGG pathway and in GO enrichment analysis of DEMs, it was revealed that expression levels for key components such as apoptosis, Wnt signaling, and p53 signaling pathway of the colorectal cancer pathway were significantly altered.

One of the limitations of our study was that we did not collect cancer samples from patients. Determining expression levels of both *CHRM3* and *EGFR* in tumor tissue obtained from individuals with colon cancer might enlighten in vivo reflections and importance of our results. Colon cancers might exhibit different patterns of genetic expressions and other colon cancer cell lines could be investigated for specific miRNA and *CHRM3* expression as well. Although SNU-C4 and H508 cells are known as colon cancer cells, since these cells originate from different parts of the colon, different gene interactions may have played a role in these cell lines. By suppressing the *CHRM3* gene in the H508 cell line with siRNA, the difference in miRNA pattern in both H508 and SNU-C4 should also be examined. Another limitation is that we did not measure all possible miRNAs reported as related to oncogenesis since their numbers increased exponentially each day.

These findings reflected that *CHRM3*-dependent oncogenetic pathways might be in charge of colon cancer. The expression of *CHRM3*-dependent hsa-miR-1246 and hsa-miR-522-3p could play a critical role in tumor development via bile acid-related genes in colon cancer. We suggest that miRNA expression profile of each individual colon cancer tissue is a unique digital signature.

## Figures and Tables

**Table 1.** miRNA Alterations in Response to ST Treatment in the Colon Cancer Cell Lines

miRNA	H508-ST		SNU-C4-ST	
	*P*	Fold Regulation	*P*	Fold Regulation
hsa-miR-545-5p	.303391	−1.3013	.238704	−1.3059
hsa-miR-629-5p	.349764	1.7291	.474122	3.9313
hsa-miR-30a-5p	.334359	1.4914	.072444	−3.4862
hsa-miR-129-5p	.047729	3.1311	.940399	−1.1057
hsa-miR-30c-5p	.000125	1.7291	.035600	−6.0839
hsa-miR-30d-5p	.129525	1.5476	.093849	−3.4542
hsa-miR-210-3p	.934176	1.0187	.191448	−2.4033
hsa-miR-224-5p	.000027	4.0652	.202437	−4.352
hsa-miR-30b-5p	.029118	1.4175	.042316	−4.9417
hsa-miR-130a-5p	.149877	5.4014	.555262	−1.1316
hsa-miR-30e-5p	.014071	−1.7942	.046762	−2.8979
hsa-miR-522-3p	.000128	12.4092	.170822	−1.5351
hsa-miR-668-3p	.663248	1.5333	.087756	−2.2372
hsa-miR-1283	.09805	1.4373	.254516	−1.2968
hsa-miR-147b-3p	.030618	−1.7736	.462489	−1.4028
hsa-miR-885-3p	.016729	−2.6574	.764446	−1.514
hsa-miR-1226-3p	.70213	1.0377	.404195	−2.7606
hsa-miR-1238-3p	.911485	1.1434	.162662	−1.321
hsa-miR-1246	.000005	39.0342	.043789	−3.6175
hsa-miR-1262	.350783	−1.4743	.254516	−1.2968
hsa-miR-103a-2-5p	.288971	−1.4811	.139479	−2.171
hsa-miR-1249-3p	.840888	1.2058	.197113	−2.2842
hsa-miR-222-5p	.829949	1.5192	.198002	−1.521
hsa-miR-34b-5p	.358977	1.192	.03941	−3.014
hsa-miR-4297	.873184	1.0842	.550125	−1.1109

miRNA, micro RNA; ST, sodium taurolithocholate;

**Table 2.** The Result of KEGG and GO Enrichment Analysis for the 10 Differentially Expressed miRNAs (*P* <.05)

KEGG Pathway	*P*	#genes	#miRNAs
Fatty acid biosynthesis	0	3	3
Fatty acid metabolism	0	12	4
Lysine degradation	0	17	5
Oocyte meiosis	2.06E-13	35	4
Mucin-type O-glycan biosynthesis	6.80E-12	8	3
Ubiquitin-mediated proteolysis	1.00E-10	40	3
p53 signaling pathway	7.95E-07	29	6
Pathways in cancer	6.16E-06	72	4
Viral carcinogenesis	2.78E-05	38	3
Protein processing in endoplasmic reticulum	.000234898	45	4
**GO Category**			
Post-translational protein modification	0	42	4
Protein binding transcription factor activity	0	118	5
DNA metabolic process	0	128	5
Enzyme regulator activity	0	145	5
Cellular protein metabolic process	0	106	5
Nucleic acid binding transcription factor activity	0	176	6
Transcription, DNA-templated	0	376	6
Transcription initiation from RNA polymerase II promoter	0	57	6
Blood coagulation	0	96	6
Cell death	0	169	6

GO, gene ontology; KEGG, Kyoto Encyclopedia of Genes and Genomes.
